# Array-based identification of common DNA methylation alterations in ulcerative colitis

**DOI:** 10.3892/ijo.2011.1283

**Published:** 2011-12-06

**Authors:** KEI KOIZUMI, SERGIO ALONSO, YUICHIRO MIYAKI, SHINICHIRO OKADA, HIROYUKI OGURA, NORIHIKO SHIIYA, FUMIO KONISHI, TOSHIKI TAYA, MANUEL PERUCHO, KOICHI SUZUKI

**Affiliations:** 1 First Department of Surgery, Hamamatsu University School of Medicine, 1-20-1 Handa-yama, Higashi-ku, Hamamatsu, Shizuoka 431-3192, Japan; 2 Institute of Predictive and Personalized Medicine of Cancer (IMPPC), Carretera de Can Ruti S/N, 08916 Badalona, Spain; 3 Department of Surgery, Saitama Medical Center, Jichi Medical University, 1-847 Amanuma-cho, Omiya-ku, Saitama 330-8503; 4 Agilent Technologies, 9-1 Takakura-cho, Hachioji, Tokyo 192-8510, Japan; 5 Sanford-Burnham Medical Research Institute, Tumor Development Program, 10901 North Torrey Pines Road, La Jolla, CA 92037, USA; 6 Catalan Institution for Research and Advanced Studies (ICREA), Pg. Lluís Companys 23, 08010 Barcelona, Spain

**Keywords:** ulcerative colitis associated carcinogenesis, methylation profiling, field cancerization, methylation sensitive amplified fragment length polymorphism, microarray, MS-AFLP

## Abstract

Patients with long-standing ulcerative colitis (UC) have higher risk of developing colorectal cancer. Albeit the causes remain to be understood, epigenetic alterations have been suggested to play a role in the long-term cancer risk of these patients. In this work, we developed a novel microarray platform based on methylation-sensitive amplified fragment length polymorphism (MS-AFLP) DNA fingerprinting. The over 10,000 *Not*I sites of the human genome were used to generate synthetic primers covering these loci that are equally distributed into CpG rich regions (promoters and CpG islands) and outside the CpG islands, providing a panoramic view of the methylation alterations in the genome. The arrays were first tested using the colon cancer cell line CW-2 showing the reproducibility and sensitivity of the approach. We next investigated DNA methylation alterations in the colonic mucosa of 14 UC patients. We identified epigenetic alterations affecting genes putatively involved in UC disease, and in susceptibility to develop colorectal cancer. There was a strong concordance of methylation alterations (both hypermethylation and hypomethylation) shared by the cancer cells of the CW-2 cell line and the non-cancer UC samples. To the best of our knowledge, this work defines the first high-throughput aberrant DNA methylation profiles of the colonic mucosa of UC patients. These epigenetic profiles provide novel and relevant knowledge on the molecular alterations associated to the UC pathology. Some of the detected alterations could be exploited as cancer risk predictors underlying a field defect for cancerization in UC-associated carcinogenesis.

## Introduction

Ulcerative colitis (UC) is a chronic disease characterized by inflammation of the mucosa and submucosa of the large intestine. The duration and extent to which a patient suffers from UC are directly proportional to a propensity for colorectal cancer (1,2). Ulcerative colitis patients have elevated risk of colorectal cancer. This risk has been estimated to be 2% after 10 years, 8% after 20 years and 18% after 30 years of disease (3). Unlike sporadic colorectal cancers that typically follow the adenoma-carcinoma pathway, UC-associated colorectal tumors progress from areas of dysplastic mucosa, following what it has been termed the dysplasia-carcinoma pathway. These tumors typically develop as flat lesions, which in many instances are difficult to detect, especially in areas of active inflammation (4–8). Owing to improvements in UC treatment this disease is rarely lethal *per se*, and the associated mortality is largely determined by the incidence of colorectal cancer. As of today, very little is known about the molecular basis of the UC-associated colorectal cancer. This highlights the urgent need to determine the early genetic and epigenetic alterations germane to the UC-associated carcinogenesis (9). Discerning the mechanisms underlying UC-associated colorectal cancer will facilitate the identification of markers for predisposition of the UC-associated carcinogenesis, which in turn might improve the prognosis and treatment of UC patients.

In several diseases characterized by chronic inflammation, such as chronic hepatitis and *Helicobacter pylori* infection chronic gastritis, aberrant DNA methylation of promoter-associated CpG islands is associated with transcriptional inactivation of tumor suppressor genes (10). In addition, aberrant DNA methylation has been observed not only in cancer tissues but also in tissues that appear to be histologically normal (11–13), suggesting that some cancers arise from regions containing certain epigenetic alterations that may be shared with an accompanying cancer. This phenomenon is exemplified by the ‘field cancerization’ caused by carcinogen exposure (14). Molecular alterations such as p16 (15) and p14 hypermethylation (16), were more frequently observed in non-neoplastic epithelium of UC patients with neoplasia than in those without neoplasia, suggesting that these molecular alterations may be exploited as markers for identifying individuals with UC at increased risk of neoplasia. Chronic inflammation has been reported to associate with high levels of CpG island hypermethylation, perhaps as a result of increased cell turnover, and that age-related methylation marks (and may lead to) the field defect that reflects acquired predisposition to colorectal neoplasia (17). Fujii *et al*, reported that ER gene methylation may be potentially useful for identifying individuals at increased risk of neoplasia among patients with long-standing and extensive UC (18). In addition, global DNA hypomethylation in the rectal mucosa of patients with UC has been proposed to be the result of the increased proliferative activity triggered by the active inflammation, and that this epigenetic change might predispose these individuals to develop colorectal neoplasm (19). The genomic extent of methylation alterations in UC mucosa, however, remains to be accurately defined. In this regard, genome wide analyses of methylation alterations provide an effective strategy to elucidate the influence of epigenetic alterations on field cancerization.

Methylation sensitive amplified fragment length polymorphism (MS-AFLP) is a fingerprinting technique developed by Yamamoto *et al* as a tool to analyze DNA methylation in hundreds of loci simultaneously (20,21). MS-AFLP is based on the differential PCR amplification of methylated vs. unmethylated *Not*I sites. We employed this technique to demonstrate that the accumulation of DNA methylation alterations in human colorectal and gastric cancers occurs in a gradual fashion (22), and that this accumulation associates with aging and precedes genomic damage (23). More recently, we developed a fluorescence-labeled MS-AFLP technique for the detection of hyper- and hypomethylation alterations using a fluorescence-detecting electrophoresis apparatus (24). One advantage of the MS-AFLP over similar approaches is that approximately half (44%) of the *Not*I recognition sites are located in or adjacent to CpG islands, while the rest (56%) are located outside the CpG islands. This distribution enables to detect both DNA hypermethylation, generally occurring closer to CpG islands, and DNA hypomethylation, generally affecting repetitive elements and single copy loci outside CpG islands (25). Thus, MS-AFLP provides unbiased insight into the complex picture of hypermethylation and hypomethylation alterations, both of which are known to be associated with cancer (26). In its original format, however, MS-AFLP was limited by the number of bands than can be resolved in a single electrophoretic gel, typically less than 100. This limitation could be partly overcome by employing several primer combinations that amplify different subsets of the *Not*I-*Mse*I fragment, but this solution is still insufficient to analyze the nearly 10,000 *Not*I sites of the human genome. A previous attempt to increase its throughput by applying MS-AFLP to a DNA microarray hybridization demonstrated the feasibility of the approach (27). This methodology, however, required the cloning and sequencing of individual MS-AFLP fragments before printing them onto the arrays, two very labor-intensive steps that represented an important hurdle for scaling up the throughput of the MS-AFLP microarrays.

In this study, we have designed a novel MS-AFLP array-based platform containing probes consisting of 60-mer-oligonucleotides, which cover the sequences adjacent to all the *Not*I sites identified in the human genome sequence. Using this platform, we have identified methylation alterations in non-neoplastic UC epithelia. Our results describe for the first time the genome-wide methylation profile of UC mucosa samples, and provides clues for the increased risk for developing colorectal cancer.

## Materials and methods

### Cell culture, patients and tissues

The human colon cancer cell line CW-2 (RCB0778) was provided by the RIKEN Cell Bank (RCB, Tsukuba, Japan). CW-2 cells were cultured in Roswell Park Memorial Institute 1640 (RPMI-1640) medium supplemented with 10% fetal bovine serum (FBS), 10,000 U/ml penicillin G, 10 mg/ml streptomycin sulfate and 25 μg/ml amphotericin B. The cells were maintained at 37°C in a humidified incubator with 5% CO_2_.

Fourteen non-neoplastic UC epithelia biopsies (UCM) were obtained from patients who had undergone surgery at Jichi Medical University Saitama Medical Center and Jichi Medical University hospital. In addition, 11 normal colon mucosal tissues from healthy volunteers were collected and used as controls. The study design was approved by the Institutional Review Board (IRB) of Hamamatsu University School of Medicine and Jichi Medical University.

### Design of the methylation sensitive amplified fragment array

To design the MS-AFLP array, we retrieved all the 80-bp upstream and downstream sequences adjacent to every one of the 9,654 *Not*I sites previously mapped on the Build 36 of the human genome sequence obtained from the NCBI. These sequences were employed to design 19,308 60-mer microarray probes using OligoArray 2.0, a program that designs specific oligonucleotides at the genomic scale (28). This process generated a set of 19,308 probes, two probes (one upstream and one downstream) per every *Not*I site in the human genome sequence. Using the same process, 2,200 additional methylation-insensitive probes within *Mse*I-*Mse*I fragments were designed to control for copy number alterations. All these oligonucleotides were synthesized in duplicate onto array slides by an ink-jet oligonucleotide synthesizer apparatus at Agilent Technologies. The MS-AFLP array comprises a total number of 43,016 synthesized probes: 19,308×2 methylation sensitive probes, plus 2,200×2 methylation-insensitive probes, and approximately two thousand additional features required for the array scanning and quality control. *Not*I sites are associated to DNA regions with a high GC content, which are also rich in repetitive elements. Mapped *Not*I sites (8.8%) occur within or adjacent to repetitive sequences, in particular AluY elements. Hence, some *Not*I-associated probes are unavoidably located inside repetitive elements. To identify those probes complementary to repetitive elements, every probe was re-mapped on the entire human genome sequence allowing up to one mismatch. Of the 19,308 probes, 18,086 (93.7%) were unique in the genome while 1222 (6.3%) mapped in two or more locations (896 probes mapping in 2–10 loci, 130 probes mapping in 11–100 loci, 110 probes mapping in 101–1,000 loci, 80 probes mapping in 1,001–10,000 loci, and 6 probes mapping in >10,000 loci). Therefore, the MS-AFLP array design allows the determination of methylation alterations in unique loci, moderately repetitive DNA elements and highly repetitive DNA elements. The MS-AFLP arrays employed in this study were printed at Agilent Technologies facilities at Hachioji (Tokyo 192–8510, Japan), under a collaborative arrangement.

### Preparation, labeling and hybridization of DNA samples for MS-AFLP arrays

Genomic DNA was isolated by QIAamp DNA Mini Kit (Qiagen, Hilden, Germany). The initial steps of the MS-AFLP were performed as previously described (20). Briefly, 1 μg of genomic DNA was digested overnight with 5 U of methylation-sensitive *Not*I (Promega, Madison, WI, USA) and 2 U of methylation-insensitive *Mse*I (NE Biolabs, Beverly, MA, USA) at 37°C. Two pairs of oligonucleotides were annealed overnight at 37°C to generate *Not*I (5′-CTCGTAGACTGCGTAGG-3′ and 5′-GGCCCCTACGCAGTCTAC-3′) and *Mse*I (5′-GACGATG AGTCCTGAG-3′ and 5′-TACTCAGGACTCAT-3′) specific adaptors. The digested DNA was ligated to 1.25 μl each of 5 pmol/μl *Not*I and 50 pmol/μl *Mse*I adaptor using 1 U of T4 DNA ligase (Promega) overnight at 16°C. The adaptor-ligated template DNA was amplified by PCR using *Not*I primer (5′-GACTGCGTAGGGGCCGCG-3′) and *Mse*I primer (5′-GATGAGTCCTGAGTAA-3′). The PCR mixture consisted of 6 ng of *Not*I primer, 30 ng of *Mse*I primer, 0.25 mM dNTP, and 1.5 U of AmpliTaq DNA polymerase (Applied Biosystems, Foster City, CA, USA) in a final volume of 20 μl. The PCR started at 72°C for 30 sec and 94°C for 30 sec, followed by 35 cycles of 94°C for 30 sec, 52°C for 30 sec, and 72°C for 2 min. The final extension was performed for 10 min at 72°C. The reactions were then kept at 10°C until the amplified DNA fragments were isolated using a QIA PCR Clean-up kit (Qiagen). DNA was eluted into 50 μl of elution buffer.

Prior to hybridization on the MS-AFLP arrays, the DNA samples were labeled as previously described (27). Briefly, fluorescently labeled fragments were prepared using the Bioprime labeling system (Invitrogen). Each sample of PCR-amplified DNA (50 ng/2.5 μl) was mixed with 5 μl of water and 5 μl of random primer mix solution. The mixtures were boiled at 100°C for 2 min, quickly placed on ice for 1 min, and briefly centrifuged for 10 sec. Then 1 μl of either CY5 mix solution (1.56 mM each of dGTP, dATP and dTTP, 0.22 mM dCTP, and 0.11 mM Fluorolink CY5-dCTP) or CY3 mix solution (1.56 mM each of dGTP, dATP, and dTTP, 0.22 mM dCTP, and 0.11 mM Fluorolink CY3-dCTP) was added. Fluorolink CY5-dCTP and CY3-dCTP were purchased from Amersham-Pharmacia. Klenow fragment of *E. coli* DNA polymerase was then added to a final concentration of 0.8 U per μl. The mixtures were incubated at 37°C for 1 h before adding 2 μl of stop solution (0.5 M EDTA) to terminate the reaction. The CY5 and CY3 fluorescently labeled DNA fragments were separated from the unincorporated dNTPs by filtration through Microcon YM-30 columns (Millipore, Bedford, MA, USA). Each sample was reconstituted with 1X TE (pH 8.0) to a final volume of 37 and 2 μl of each sample was taken to determine the yield of labeled genomic DNA and the specific activity after labeling and clean-up. Exposure of samples to light was minimized during all experimental procedures.

The Cy3 and Cy5 labeled DNA samples were mixed in a siliconized tube with 70 μl of Agilent 2X Hi-RPM buffer (Agilent, Santa Clara, CA, USA). The mix was heated at 95°C for 3 min and centrifuged at 6000 × g for 1 min to collect the sample at the bottom of the tube. Hybridization sample mixture (110 μl) was applied slowly to the gasket slide into the Agilent SureHyb chamber base. Then, one microarray slide was placed onto the gasket slide, with the active side facing down. The SureHyb chamber was covered onto the slides, and the clamp assembly was slid onto both pieces. The assembled slide chamber was placed in a rotator rack inside a hybridization oven and rotated at 20 rpm and hybridized at 65°C for 40 h. After hybridization, array slides were washed with Oligo aCGH wash buffer 1 at room temperature for 5 min and Oligo aCGH wash buffer 2 at 37°C for 1 min. To prevent Cy5 degradation by ozone, the slides were washed with acetonitrile for 30 sec and then with stabilization and drying solution for 30 sec. The arrays were scanned using an Agilent G2565BA DNA Microarray Scanner.

### MS-AFLP array data analysis

After scanning, data extraction was conducted using the Feature Extraction software version A.10.5.1.1 (Agilent Technologies). GeneSpring version 10 (Agilent Technologies) was used to export the array data. For the MS-AFLP array data analysis, an *ad hoc* pipeline was developed in R (29). The background corrected signals were used to calculate the log2 ratio of the CY5 vs CY3 channel for every array feature. The log2 ratios were subsequently normalized by LOWESS method (locally weighted scatterplot smoothing), using the information from all the features in the array. Then, the normalized log2 ratios from the 19,308 methylation-sensitive probes were extracted from the dataset. Since these probes are synthesized in duplicate in the array, two independent log2 ratio values were obtained for each probe. These values were combined into a single value by calculating their weighted mean, where the weights were given by the total intensity of the replicates in both the CY3 and CY5 channels. Finally, the probes within the 30% lowest intensity in both the CY3 and CY5 channels in both replicates, likely representing cross-hybridization noise, were excluded from the subsequent analyses. The thresholds for hypermethylation and hypomethylation were set at log2 ratio < -1 and log2 ratio > 1.

### Bisulfite sequencing

Bisulfite sequencing was performed as previously described (30). Genomic DNA was modified with EpiTect Bisulfite Kit™ (Qiagen). Bisulfite-modified DNA was analyzed by PCR with primers specifically designed to amplify the *Not*I-containing products of interest. The primers and annealing temperature for the bisulfite sequencing were: spot a (*Not*I site no. 5391, primers 5′-TGTTATAGGGATGGA TTTTGTTAT-3′ and 5′-CCACCCCTATATCATACCCT-3′, annealing temperature: 56°C); spot b (*Not*I site no. 2627, primers 5′-GGTGGTTTGTTTTTTTGAAGA-3′ and 5′-AACCTATAT CAAAACACCCCC-3′, annealing temperature 50°C); spot c (*Not*I site no. 6105, primers 5′-GAGTGATTGAATTAAGA AGAGTAAAA-3′ and 5′-AAACTCAAACCCAATTAATT AAA-3′, annealing temperature 46°C); spot d (*Not*I site no. 374, primers 5′-TGTGTTAATATTTGGGGGTAGAG-3′ and 5′-TACTTCTACCCTAACCTCCCCT-3′, annealing temperature 53°C); spot e (*Not*I site no. 563, primers 5′-GTG TTTTAGGAGGATTTGGG-3′ and 5′-AAAAAATACCCC TATCTCACCTC-3′, annealing temperature 51°C); and spot f (*Not*I site no. 3511, primers 5′-GTTTTTATTTGAGGGGGA ATG-3′ and 5′-TACTCTTAACCTAACTCACCCCC-3′, annealing temperature 51°C). PCR amplifications were carried out in 20 μl reaction mixtures containing 1X PCR buffer, 0.25 mM (each) dNTP mix, 0.025 U/μl HotStarTaq Plus DNA Polymerase (Qiagen) and 0.5 μM each primer. The PCR conditions were 95°C for 5 min, followed by 38 cycles of 95°C for 45 sec, then 45 sec at the specific annealing temperature, 72°C for 1 min, and then 72°C for 10 min. PCR products were purified by EXOSAP-IT (USB Corp., Cleveland, OH, USA), and cloned into the pGEM-T Easy Vector (Promega). Recombinant plasmids were sequenced with an ABI PRISM 3100 Genetic Analyzer (Applied Biosystems) using SP6 (5′-TATTTAGGTGACACTATAG-3′) and T7 (5′-TAA TACGACTCACTATAGGG-3′) primers. The methylation status of each CpG site was determined by sequencing, as unmethylated cytosines are converted into thymines after the bisulfite treatment and PCR amplification, whereas methylated cytosines remain unaltered.

### Statistical analysis

Statistical analyses were performed using the R statistical environment (29). Fisher’s exact was used to examine associations between two categorical variables. Continuous variable comparisons between two groups were performed with the Student’s t-test for those variables following a normal distribution, or with the non-parametric Mann-Whitney-Wilcoxon test for those variables that do not follow a normal distribution. Normality was assessed using the Shapiro-Wilk test. The level of statistical significance was set at P<0.01, unless otherwise specified. Holm’s multi-hypothesis testing correction was applied when appropriate (31). Hierarchical analysis and clustering analysis were performed using MeV (32). Gene category enrichment analyses were performed using DAVID (33,34), which employs a more robust variation of the Fisher’s exact test, termed EASE score (35). To account for the bias due to the partial gene representation in the MS-AFLP array, all the gene enrichment analyses were performed using the list of the genes present in the array as a background, instead of the total number of genes in the human genome.

## Results

### Set-up and testing the MS-AFLP array

**Figure 1:**
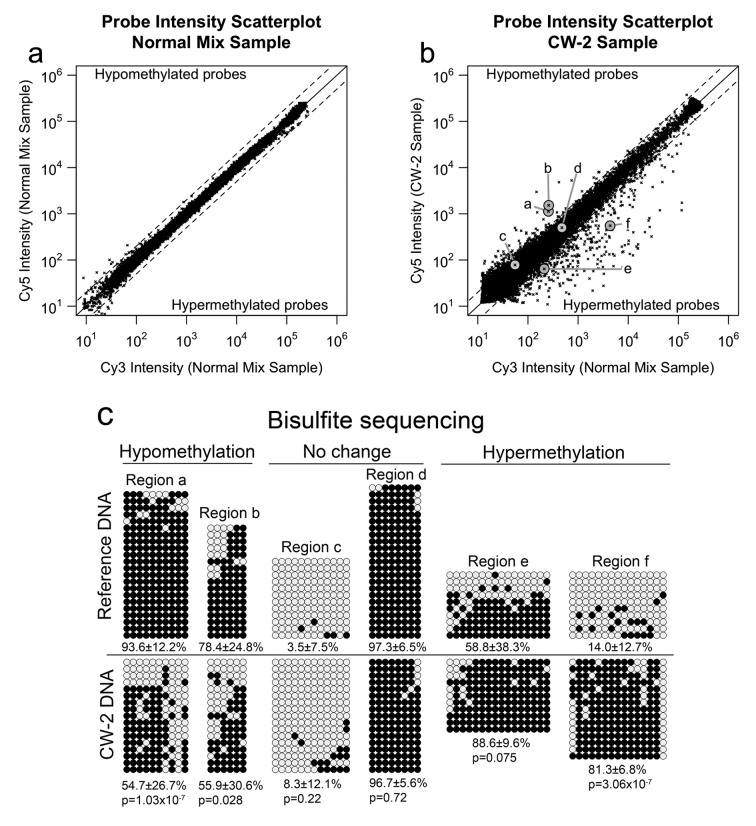
Logarithmic scatterplots of the self-self hybridization (a) and the CW-2 (b) MS-AFLP array experiments. In both graphs, the solid diagonal black line indicates the log2 ratio = 0 (no change). The dashed lines indicate log2 ratio = 1 and log2 ratio = −1, used as thresholds for hypomethylation and hypermethylation cells, respectively. Six spots were selected from the CW-2 MS-AFLP array: two hypomethylated (spots a and b), two with no change (spots c and d) and two hypermethylated (spots e and f). The methylation of the *Not*I sites associated to these spots was analyzed by bisulfite sequencing (c). Every row of circles represents the sequence of an individual clone. Black circles represent metyhylated CpG sites while white circles represent unmethylated CpG sites. For every region, the methylation fraction was calculated by averaging the methylation level of every individual clone. Statistical significance of the methylation fraction differences was analyzed by Wilcoxon rank sum test.

To estimate the false-positive rate of the MS-AFLP array we performed a self-self hybridization experiment using a mixture of DNAs obtained from the colonic mucosa of 11 healthy volunteers (see Materials and methods). This mixture of DNAs was used as reference sample in all the subsequent MS-AFLP arrays presented in this study. The results are shown in the scatter plot of Fig. 1a. In this plot, the logarithmic x-axis and y-axis represent the average signal in the Cy3 and Cy5 channels, respectively. The no-change line (log2 ratio = 0) is in solid black. The diagonal dashed lines indicate the alteration calling thresholds, set at log2 ratio > 1 for hypomethylation and log2 ratio < -1 for hypermethylation. According to the self-self hybridization experiment, the MS-AFLP array exhibited a 0.12% of false positives mostly in the low-intensity subset of probes, more susceptible to spurious fluctuations due to probe cross-hybridization. Applying a very stringent floor filter excluding the 30% lowest-intensity probes, the false positive rate was reduced to <0.015%. This filter was applied by default in all subsequent MS-AFLP array analyses.

The colon cancer cell line CW-2 was selected to determine the capabilities of the array-based MS-AFLP to detect DNA methylation alterations. CW-2 is a colorectal cancer cell line derived from a 60-year-old female patient with lymph node metastases. The cells display microsatellite instability (MSI) due to an inactivating frameshift mutation in *hMLH1*. This alteration is fixed in homozygosis due to copy neutral LOH affecting the 62-Mb distal region of the 3p chromosome, where *hMLH1* is located (data from The Cancer Genome Project of The Wellcome Trust Sanger Institute). Except for the loss of a 13-Mb region of chromosome X, CW-2 cells harbor very few copy number alterations (CNAs), and they are mostly diploid with 84% of cells harboring a normal content of 46 chromosomes (information from the RIKEN BioResource Center).

The result of the MS-AFLP array analysis of CW-2 DNA relative to a pooled sample of normal colon tissue DNAs is shown in Fig. 1b. To validate the methylation changes detected by the MS-AFLP array, we selected six probes representing two hypomethylated *Not*I sites (a and b), two non-altered *Not*I sites (c and d) and two hypermethylated *Not*I sites (e and f). These loci were analyzed by bisulfite genomic sequencing, the current gold standard technique to analyze DNA methylation, using the primers indicated in Materials and methods. The results obtained by bisulfite sequencing (Fig. 1c) confirmed the results from the MS-AFLP array in all the analyzed loci, demonstrating the capability of the MS-AFLP array to detect DNA methylation alterations. In addition, no association between the chromosomal location of the DNA methylation alterations and the copy number variations reported in CW-2 was observed (data not shown), which further supports the validity of this platform to accurately detect somatic epigenetic changes even within chromosomal regions that had undergone moderate copy number alterations.

The MS-AFLP array analysis of the genome of CW-2 DNA revealed 500 probes (3.75%) with log2 ratio below the hypermethylation threshold, and 129 probes (0.95%) with log2 ratio above the hypomethylation threshold (Fig. 1b). Since this cell line is derived from a female patient, the data from the Y chromosome probes was removed from the analysis. Due to design strategy constrains, the MS-AFLP array contains 94 probes with mononucleotide repeats of 9 or more nucleotides (polyN). Shortenings and expansions of mononucleotide repeats are the archetypical feature of the MSI phenotype resulting from defects in the mismatch repair mechanisms (36). Since CW-2 is an MSI cell line, we investigated whether the polyN-containing probes exhibited a particular behavior. Out of the 94 probes with polyN sequences, 8 where excluded due to low intensity in both the Cy3 (reference sample) and Cy5 (CW-2 sample) channels. Of the remaining 86 probes, 17 (19.8%) exhibited a log2 ratio below the hypermethylation threshold (i.e., significantly hypermethylated), a much higher frequency than that of probes without polyN (483/13015, 3.7%). None of the polyN-containing probes exhibited log2 ratios above the hypomethylation threshold (i.e., significantly hypomethylated). Hence, there was a very strong association between the presence of mononucleotide repeats in the probe sequences and the reduction in their signal intensity in the CW-2 sample, resulting in a negative log2 ratio (OR=6.6, 95%CI=3.6–11.5, P=1.3×10^−8^ Fisher’s exact test). While the possibility that some of these probes indicate actual methylation alterations can not be discarded, it seems more plausible that the reduction of signal intensity is a consequence of microsatellite shortenings and expansions in the genome of CW-2 cells, resulting in loss of perfect complementarity between the MS-AFLP amplicons and the MS-AFLP array probes. Therefore, to avoid miss-interpretation errors, polyN-containing probes were not considered for the scoring of alterations in the subsequent analysis of CW-2 MS-AFLP array. Of the remaining 13,395 probes, 482 (3.6%) indicated hypermethylation and 128 (0.96%) indicated hypomethylation.

The majority of the alterations were detected by probes mapping in a single location (558/610, 91.5%) or in 2–10 locations (47/610, 7.7%). Only five of the altered probes mapped in >10 locations in the genome, all of them within repetitive sequences (data not shown). We also investigated whether DNA methylation alterations were more frequently detected in *Not*I sites located close to gene transcriptional start sites (TSS), where presumably these alterations might be associated to transcriptional variations. Since the TSS is not well defined for all the annotated human genes, we considered a promoter-containing region the 10-kb region surrounding the annotated 5’ end of the genes (5 kb upstream and 5 kb downstream). In CW-2, both hypermethylation and hypomethylation alterations were actually more frequent in the *Not*I sites located outside of these promoter-related regions. The negative association of hypermethylation alterations with the 5′ regions exhibited an odds ratio (OR) of 0.80 (95% CI 0.66–0.96, P=0.018 Fisher’s exact test). The negative association of hypomethylation alterations with the 5′ regions exhibited an OR=0.62 (95% CI 0.43–0.89, P=0.008). This observation is in agreement with recent findings showing that in colon cancer most DNA methylation alterations occur not in promoters or in CpG islands, but in ‘shore’ sequences up to 2 kb distant (25). A list of the *Not*I sites with methylation alterations in CW-2, their genomic location, and the associated genes is available upon request.

Using the database for annotation, visualization and integrated discovery v6.7 (DAVID) (33,34) we analyzed the enrichment of gene families among the hypermethylated or hypomethylated genes versus the list of genes present in the array. For hypermethylation, this analysis revealed a highly significant overrepresentation of genes encoding transcriptional factors (GOterm 0003700, n=49, fold enrichment, 1.86; EASE score, 1.14×10^−5^), in particular, transcriptional factors with homeobox domains (Interpro IPR001356, n=25, fold enrichment, 3.32; EASE score, 1.9×10^−7^). In addition, forty-two of the hypermethylated genes are involved in developmental processes (fold enrichment, 1.99; EASE score, 1.74×10^−5^). Among the hypomethylated genes, we found an enrichment of genes involved in epithelial cell development (GO term 0060429, n=6, fold enrichment, 4.37; EASE score, 0.01).

### DNA methylation alterations in the colonic mucosa of UC patients

Once the MS-AFLP array was validated in the CW-2 colorectal cancer cell line, we analyzed 14 colonic mucosa samples obtained from UC patients (UCM). Table I shows the clinicopathological features of the patients recruited in this study and the frequency of DNA methylation alterations detected by the MS-AFLP array analyses. No association was found between patient age, duration of disease or inflammation grade and the incidence of methylation alterations. The analysis of the data from all the UCM samples together revealed a negative association between methylation alterations and the 5′ region of genes (OR=0.84, P=0.0013, 95%CI 0.75–0.93 for hypermethylation, and OR=0.78, P=7.5×10^−5^, 95%CI=0.69–0.88 for hypomethylation. Fisher’s exact test).

### Identification of frequently altered loci

**Figure 2:**
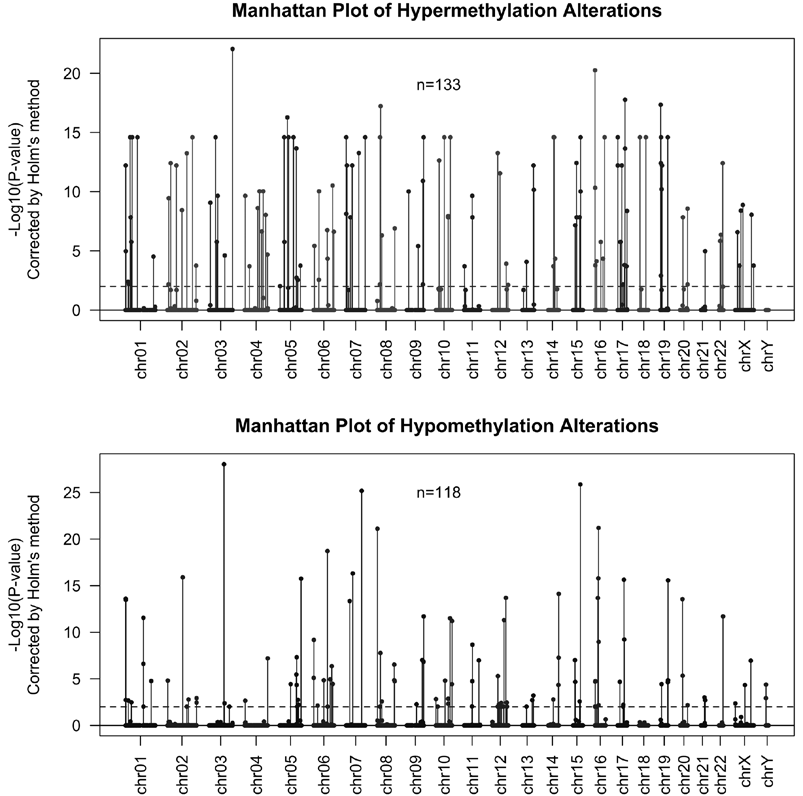
Manhattan plots of the frequency of hypermethylation alterations (upper graph) and the frequency of hypomethylation alterations (lower graph) in the 14 analyzed UC samples. The p-values were calculated by a binomial test based on the frequency of alterations empirically obtained from every individual sample, under the null hypothesis that every probe had the same probability of being altered within a sample. P-values were subsequently corrected for multihypothesis testing by Holm’s method. The dashed horizontal line represents the applied statistical significance level, set at P=0.01.

To identify the probes altered in the UCM samples with a statistically significant frequency, we applied a binomial test based on the frequency of alterations empirically obtained from every individual sample, under the null hypothesis that every probe had the same probability of being altered within a sample. The level of statistical significance was set at P<0.01 after correction for multi-hypothesis testing by the Holm method (31). Results are shown in Fig. 2. This analysis rendered 133 probes indicating hypermethylation in 3–12 of the 14 samples (frequently hypermethylated set), and 118 probes indicating hypomethylation in 3–14 of the 14 samples (frequently hypomethylated set). Twelve probes were common to both hypermethylated and hypomethylated sets, that is, hypermethylated in three or more samples and hypomethylated in three or more different samples.

The frequently hypermethylated probe set was enriched in genes participating in transcriptional processes, i.e., encoding transcriptional factors and transcriptional modulators and proteins with RNA-binding function, and genes involved in signal transduction, such as protein kinases and SH2 domain-containing proteins (data not shown). Interestingly, the second most frequently hypermethylated probe is located in the promoter of MPG, a gene that codes for the N-methylpurine-DNA glycosylase involved in the repair of DNA alkylation lesions caused by reactive oxygen and nitrogen species (RONS), which are typical products of the chronic inflammation process. In addition, six of the frequently hypermethylated genes, i.e., CCND1, COL4A2, HDAC2, AXIN2, ABL1 and GLI2, are included in the pathways in cancer map from the KEGG database (37).

### Hypomethylation associates with repetitive elements

In comparison with the frequently hypermethylated probe set, the gene enrichment analysis of the frequently hypomethylated probe set revealed a lower number of highly-significant gene categories. Of note, we found a borderline significant enrichment of genes with protein kinase C-like domains (RASGRP1, CDC42BPB and PRKCB; EASE score, 0.14; Fisher’s exact test P=0.016). Two of these genes, RASGRP1 and PRKCB, function as positive regulators of the Ras signaling pathway. The analysis also revealed a significant enrichment (fold enrichment, 8.0; EASE score, 0.05; Fisher’s exact test P=0.006) of genes associated with cell differentiation (NOG, FOXC1 and HNRNPAB).

Among the 118 frequently hypomethylated probes, seven were complementary to high copy number elements, revealing a strong association between hypomethylation and DNA repetitive elements (OR=6.3; 95%CI 2.4–13.7; P=2.1×10^−4^, Fisher’s exact test). A more extensive analysis of the 196 MS-AFLP array probes complementary to high-copy repetitive elements (>100 hits in the genome) revealed that more than half of the samples (8 out of 14) showed hypomethylation in at least one of the probes located in Alu repetitive elements. In contrast, none of these 196 probes indicated hypermethylation in any of the 14 analyzed cases. Demethylation of repetitive elements is a generally accepted surrogate of global DNA hypomethylation (26). Hence, this finding is in agreement with previous reports that global genome-wide demethylation is a common phenomenon in the inflamed colonic mucosa of UC patients (19).

### Clustering of altered loci in the colonic mucosa of UC patients

**Figure 3:**
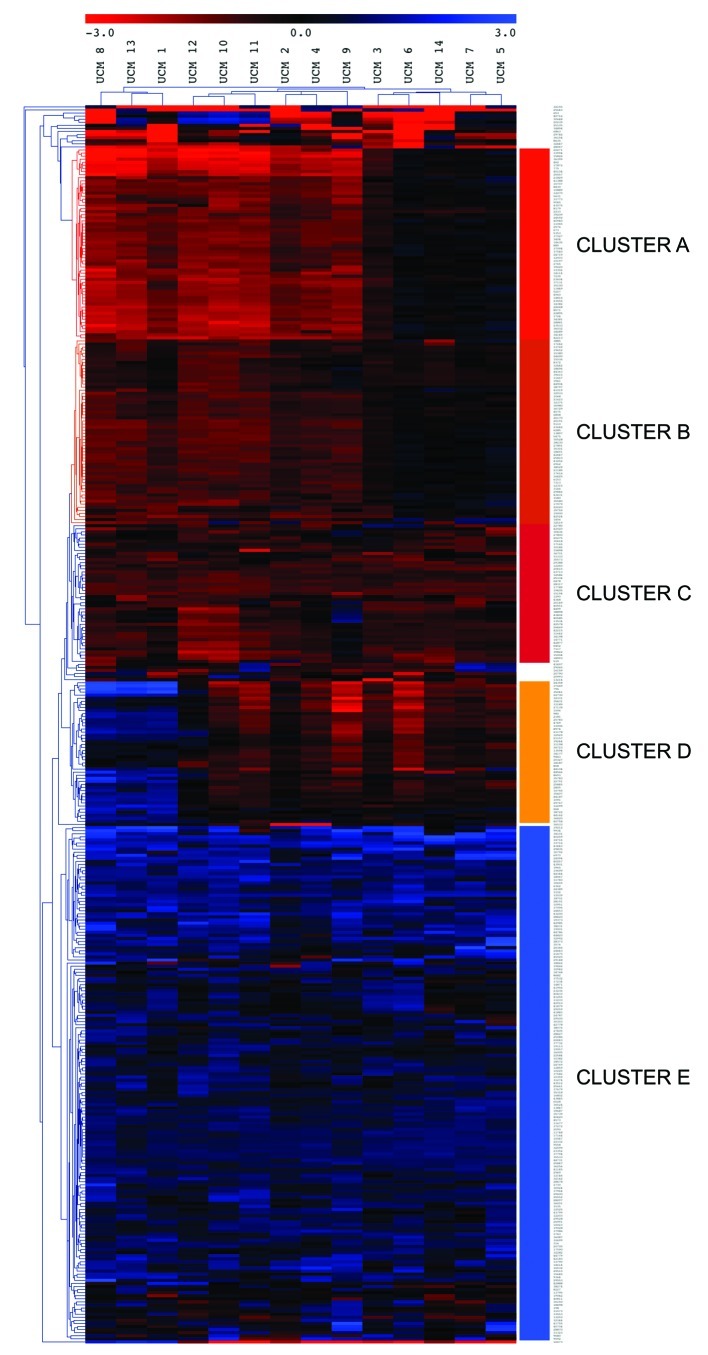
Heatmap of the methylation alterations detected by the MS-AFLP arrays. Only alterations detected in two or more of the UC samples are included in the figure. Red indicates hypermethylation (log2 ratio < −1) and blue indicates hypomethylation (log2 ratio > 1). Every column corresponds to a different sample, and every row corresponds to a different MS-AFLP array spot. Cases and spots were clustered according to the Euclidean distance using MeV. The analysis revealed 5 main spot clusters (A–E). Samples were classified in three main groups based on the methylation status of the clusters A, B and D. For more information about the genes in each cluster, see the main text.

We performed a Euclidean distance unsupervised cluster analysis with the data from all the probes hypermethylated in 2 or more samples or hypomethylated in 2 or more samples (Fig. 3). This analysis revealed 5 main gene clusters, named A–E, and three distinct groups of samples that were primarily defined by differences in the methylation status of three of these gene clusters (A, B and D). Clusters C and E were mainly hypermethylated and hypomethylated, respectively, in all the samples and exhibited lower classification power. The clustering revealed three distinctive groups of patients. A group of three cases (samples 1, 8 and 13, left of the heatmap) was characterized by strong hypermethylation of cluster A, and hypomethylation of cluster D. A second group of 6 patients (samples 2, 4, 9, 10, 11 and 12, center of the heatmap) showed hypermethylation in all clusters, but cluster E. Finally, a group of 5 patients (samples 3, 5, 6, 7 and 14, right of the heatmap) exhibited a characteristic absence of hypermethylation in clusters A and B. No obvious differences were found among the three groups of patients regarding age, gender, degree of inflammation or duration of the disease. A list of the *Not*I sites with DNA methylation alterations in these UCM samples, their genomic location, and the associated genes is available upon request.

### Concordance of epigenetic alterations in non-cancer colonic mucosa of UC patients and CW-2 colon cancer cells

We compared the alterations in *Not*I sites observed in the UCM samples with those identified in the previous analysis of the CW-2 cancer cell line. There was a strong concordance between the alterations found in the UCM samples and those found in the CW-2 cell line, both for hypermethylation and hypomethylation alterations (Table II). The detailed gene-enrichment and gene ontology analysis of these data will be published elsewhere.

## Discussion

We described a novel microarray-based platform, termed MS-AFLP array, designed to analyze DNA methylation alterations in the nearly 10,000 *Not*I sites of the human genome. This platform is based on the robust MS-AFLP technique (20) that we previously employed to show that DNA methylation alterations are gradual and that hypomethylation appears to be dominant over hypermethylation in linking epigenotype with cancer genotype and clinical outcome in sporadic gastro-intestinal cancers (22,23). Our platform possesses some unique characteristics compared to other methylation microarrays platforms. Most notably, it does not rely on the treatment of DNA with bisulfite (30). Instead, the DNA samples are digested with a combination of one methylation-sensitive (*Not*I) and one methylation-insensitive (*Mse*I) restriction enzymes, then adaptors are ligated and the fragments are amplified by PCR. Thus, the genomic distribution of probes is dictated by the distribution of *Not*I sites, which are located not only in promoter regions but also in intra- and intergenic regions.

We tested the platform in the colorectal cancer cell line CW-2, and showed the ability and accuracy of the MS-AFLP array to detect DNA methylation alterations (Fig. 1). Hypermethylated probes (482) were almost 5 times more frequent than the hypomethylated (128) probes. This finding might seem counterintuitive, since DNA hypomethylation, the almost constant companion to DNA hypermethylation in cancer, preferentially occurs in repetitive elements such as LINE-1 and Alu sequences that account for 34% of the human genome, while hypermethylation generally occurs in unique sequences, so the combined effect of these two types of alterations is an overall loss of DNA methylation. In the CW-2 MS-AFLP array context, however, it must be noted that: a) the vast majority of *Not*I sites lay not in LINE-1 or Alu elements but in unique loci, and therefore the detection is biased towards alterations occurring in single-copy elements, and b) CW-2 cells exhibit a particularly high level of global DNA methylation. In fact, this cell line ranks third in terms of LINE-1 methylation levels when compared to a panel of 13 colorectal cancer cell lines, only surpassed by the heavily methylated cell lines HCT116 and SW48 (38). The high level of hypermethylation of the CW-2 cells, however, does not seem to be attributed to the reported elevated level of hypermethylation of sporadic MSI colon cancer (39) because the cells contain a double mutation of MLH1 (a nonsense mutation fixed in homozygosis by copy neutral LOH) rather than its epigenetic silencing by hypermethylation.

Hypermethylation alterations in the tumor cell line were strongly associated to genes encoding transcriptional factors (fold enrichment, 1.86; EASE score, 1.14×10^−5^). The epigenetic silencing of transcriptional factors likely reflects a dramatic epigenetic reprogramming that globally alters the gene expression profile of the cancer cell. Many of these changes are probably associated to the malignant transformation of the primary tumor from which this cell line is derived rather than to the subsequent *in vitro* culturing conditions of CW-2 cells (40). This conclusion is based on the similarity of the findings between this tumor cell line and results with primary colon cancers (data not shown). For instance, the subgroup of transcription factors exhibiting the highest statistical significant over-representation comprised the homeobox-containing genes (IPR: IPR001356, fold enrichment, 3.32; EASE score, 2.95×10^−8^). This result confirms our previous finding, back in 1999, by MS-AFLP fingerprinting, that the homeobox-containing genes are preferential targets of methylation alterations in colon cancer (41), later confirmed by other groups (42–44).

A possible mechanism underlying the association between homeobox-containing genes and aberrant hypermethylation has been proposed recently. The two main cell memory systems involved in the maintenance of a stem cell state, i.e., the Trithorax (Trx) and Polycomb repressor complexes (PRCs), have been suggested to participate in the aberrant DNA hypermethylation found in colorectal cancers (45–47). It has been found that PRC-target genes, including the Hox gene clusters, are up to 12-fold more likely to have cancer-specific promoter DNA hypermethylation than non-targets (47) and that methylation on Lys27 of histone H3, a modification mediated by the Polycomb repressor complex 2 (PRC2), pre-marks genes for *de novo* methylation in cancer (46). This proposed mechanism, however, is insufficient to explain all the hypermethylation alterations observed in colorectal cancer cells, because many of the frequently altered genes are not polycomb targets. For instance, *hMLH1*, hypermethylated in the majority of sporadic MSI colorectal cancers and classified as one of the targets of the so-called CIMP (CpG island methylator phenotype) (39). Hence, the mechanisms that drive aberrant hypermethylation in cancer remains to date to be explained.

Among the hypomethylated loci, the most significant also were transcription factors involved in epithelium cell differentiation or proliferation, secreted proteins and proteins involved in acetylation. Of note, several homeobox-containing genes were also identified as hypomethylated (HOXB5, PHOX2A and HLX), indicating that these genes are under epigenetic regulation other than DNA hypermethylation.

In the second phase of this study, we employed the MS-AFLP array to study methylation alterations in the non-cancer colonic mucosa of UC patients. Overall, hypermethylated probes were also slightly more abundant that hypomethylated probes (difference, 0.17±0.29%; P=0.047, exact Wilcoxon signed rank test), but whereas hypermethylation occurred exclusively in unique or very low copy number sequences, hypomethylation exhibited a very strong positive association with highly repeated DNA elements, in particular with Alu repetitive elements which have been shown to undergo demethylation in colorectal cancer (48). Hence, this result supports the previous observation that global DNA hypomethylation is a common phenomenon in UC colonic mucosa (19), and shows a link between chronic inflammation and cancer in UC patients and accumulative demethylation of Alu repetitive elements (26,48).

We ranked the alterations based on their incidence, and then applied a strict threshold (P<0.01) to identify those exhibiting an incidence above the random chance. This analysis rendered 133 probes hypermethylated in 3–12 samples, associated to 111 genes, and 118 probes hypomethylated in 3–14 samples and associated to 93 genes (Fig. 2).

A significant number of the hypermethylated genes encode transcriptional factors and chromatin remodelers (KLF7, SLC2A4RG, TBX2, IRX2, GLI2, SOX8, YBX1, GABPB1, HDAC2, SIX1, RFX1, MKX, FOXD2, RUNX2, TLX2, TLX1), some already reported to be associated to colon cancer (49–51). Five of these transcriptional factors (IRX2, SIX1, MKX, TLX2, TLX1) contain a homeobox domain. In addition, there were alterations in genes that might contribute to the deregulation of cancer-associated signaling pathways. For instance, we found frequent hypermethylation of two probes corresponding to one *Not*I site located in the gene body of AXIN2. AXIN2 acts as negative regulator of canonical Wnt signaling pathway by enhancing the recruitment of the β-catenin degradation complex. Inactivating mutations and epigenetic silencing of AXIN2 have been previously reported to be involved in MSI colorectal cancer (52,53), mainly by deregulating the Wnt signaling pathway. The MS-AFLP array analysis also revealed epigenetic alterations in other members of the Wnt (WNT2, WNT3, WNT3A, WNT6 and WNT10B) and MAPK (CACNG8, EGFR, HSPA8, IKBKB, MAP2K3, PPP3CC, PRKCB RASGRP1, RRAS2 and TAB1) signaling pathways. In total, twenty-two of the frequently altered genes (ABL1, AXIN2, CCND1, COL4A2, DAPK3, EGFR, EPAS1, FLT3, GLI2, HDAC2, IKBKB, JUP, LAMA1, MSH6, PRKCB, PTEN, RUNX1T1, WNT10B, WNT2, WNT3, WNT3A, WNT6) belong to the pathways in cancer KEGG map (hsa05200). In addition, we found very frequent (78.6%) hypermethylation of the promoter region of MPG, a gene that encodes an alkylpurine DNA glycosylase responsible for the reparation of alkylated adenines and guanines. This type of DNA lesion can be caused from inflammation-associated reactive oxygen and nitrogen species (RONS). A recent study demonstrated that mice deficient for the murine MPG-homologous gene, i.e., Aag, accumulate more DNA lesions after dextran sulfate-induced colonic inflammation and are more susceptible to colon tumorigenesis (54). Hence, the finding of epigenetic alterations in MPG in UC patients suggests that the colonic mucosa of these patients are not only more exposed to the deleterious effect of the RONS generated by the inflammatory response, but also that they might suffer the impairment of the alkylated-DNA reparation mechanism, thus exacerbating the effect of these mutagens. We also found hypermethylation of the probe associated to the gene BOP1 in 36% of the UCM samples. This gene has been suggested to contribute to colorectal tumorigenesis by deregulating chromosomal segregation (55).

The concordance between many loci altered in the UCM samples and the cancer cells from the CW-2 cell line (Table II) indicates that the epithelial cells of the colonic mucosa of UC patients have undergone a number of epigenetic alterations shared with colon cancer cells. Therefore, some of these epigenetic changes may be useful as surrogate biomarkers for prediction of colon cancer development in normal colonic mucosa.

In conclusion, to the best of our knowledge, this work presents the first genomic DNA methylation profile underlying ulcerative colitis. Many of these alterations occur in genes that participate in well-established oncogenic pathways and are shared with fully malignant cells. Thus, our data support the concept of an epigenetic field for cancerization associated to the long-term risk for carcinogenesis in UC patients, by the accumulation of epigenetic alterations, followed by genetic alterations, that persist after the inflammation is treated and predispose the epithelial cells to progress into malignancy.

## Figures and Tables

**Table I:** Patient and tissue characteristics, and epigenetic alterations.

					Methylation alterations^b^		
					
Patient	Gender	Age	Disease duration	Matts’ score^a^	Hyper	Hypo	Total
UC.1	F	46	14	3.3	0.64%	0.64%	1.27%
UC.2	F	67	13	3.3	0.41%	0.14%	0.55%
UC.3	F	23	2	3.3	0.24%	0.37%	0.61%
UC.4	M	37	12	3.7	0.66%	0.31%	0.97%
UC.5	M	56	5	2.3	1.07%	0.64%	1.71%
UC.6	M	28	10	2.7	0.48%	0.46%	0.94%
UC.7	M	36	16	1.3	0.23%	0.61%	0.84%
UC.8	M	19	1.5	2.7	0.97%	1.02%	1.99%
UC.9	M	42	10	2	0.82%	0.68%	1.50%
UC.10	M	26	6	1.7	1.12%	0.72%	1.83%
UC.11	F	43	3	2.3	0.98%	0.50%	1.48%
UC.12	F	23	9	2.3	1.04%	0.34%	1.38%
UC.13	F	32	−9	3	0.87%	0.80%	1.67%
UC.14	M	35	10	2.7	0.42%	0.38%	0.81%
Mean ± SD	57% M	36.6±13.4	8.6±4.5	2.6±0.7	0.7±0.3%	0.5±0.2%	1.3±0.5%
	43% F						

a Histologically, the inflammatory activity of each specimen was classified according to the scale proposed by Matts SG (56) into the following categories: grade 1 (no inflammation), grade 2 (mild inflammation), grade 3 (moderate inflammation), grade 4 (severe inflammation).

b Percent of the MS-AFLP array spots that passed the low-intensity filter (13,515 per array) and exhibited log2 ratios below the hypermethylation threshold (hyper) or above the hypomethylation threshold (hypo). The total refers to the sum of hypermethylation and hypomethylation alterations.

**Table II:** Concordance between hypermethylation and hypomethylation alterations detected by MS-AFLP arrays in cancer cell line CW-2 and non-cancer UCM samples.

A, Hypermethylation alterations		

	In UCM	
	
	Hyper-methylated^a^	Non-hyper-methylated
In CW-2		
Hypermethylated	62	421
Non-hypermethylated	86	12946
	Odds ratio = 22.15	
	95% CI 15.48–31.56	
	Fisher’s exact test P<2.2×10^−16^	

B, Hypomethylation alterations		

	In UCM	
	
	Hyper-methylated^b^	No hyper-methylated

In CW-2		
Hypomethylated	27	102
Non-hypomethylated	91	13295
	Odds ratio = 38.63	
	95% CI 23.13–62.91	
	Fisher’s exact test P<2.2×10^−16^	

a Spots hypermethylated in 3 or more UCM samples.

b Spots hypo-methylated in 3 or more UCM samples. The total number of spots is 13,515 after filtering the 30% lowest-intensity spots from the 19,308 methylation-sensitive spots printed on the MS-AFLP array. The number of spots without changes is an over-estimation because they include other loci that were not sufficiently amplified in the respective experiments.

## Abbreviations

UC: ulcerative colitis
UCM: non-neoplastic UC mucosa
MS-AFLP: methylation sensitive amplified fragment length polymorphism
OR: odds ratio
CI: confidence interval
